# Characteristics of Medicaid Policies for Children With Medical Complexity by State: A Qualitative Study

**DOI:** 10.1001/jamanetworkopen.2022.39270

**Published:** 2022-10-31

**Authors:** Jennifer D. Kusma, Matthew M. Davis, Carolyn Foster

**Affiliations:** 1 Division of Advanced General Pediatrics and Primary Care, Department of Pediatrics, Ann & Robert H. Lurie Children’s Hospital of Chicago and Northwestern University Feinberg School of Medicine, Chicago, Illinois; 2 Mary Ann & J. Milburn Smith Child Health Outcomes, Research, and Evaluation Center, Stanley Manne Children’s Research Institute, Ann & Robert H. Lurie Children’s Hospital of Chicago, Chicago, Illinois; 3 Department of Medicine, Northwestern University Feinberg School of Medicine, Chicago, Illinois; 4 Department of Medical Social Sciences, Northwestern University Feinberg School of Medicine, Chicago, Illinois; 5 Department of Preventive Medicine, Northwestern University Feinberg School of Medicine, Chicago, Illinois

## Abstract

**Importance:**

Families of children with medical complexity (CMC) report barriers to accessing affordable coverage for the full range of services their children may need to optimize their health outcomes. Medicaid enrollment through medical need–based eligibility mechanisms can help cover these service gaps. Understanding state-by-state variation in how CMC access Medicaid may allow policy makers and pediatricians to help families navigate needed services for CMC.

**Objective:**

To clarify how eligibility and coverage for CMC differ for Medicaid beneficiaries across states with different policies and managed care penetration.

**Design, Setting, and Participants:**

This qualitative study used semistructured interviews with state Medicaid representatives from 23 states and Washington, DC, from February 1, 2020, to March 1, 2021. Enrollment pathways and coverage processes were discussed. Interviews were transcribed and content analysis was performed. Participants included Medicaid directors, a designee, or a state-identified policy leader with expertise in and/or responsibility for child-focused programs.

**Exposures:**

State variation in Medicaid eligibility and delivery policies.

**Main Outcomes and Measures:**

Eligibility pathways and coverage mechanisms for CMC in each state.

**Results:**

A total of 43 informants from 23 states and Washington, DC, participated, which permitted data collection regarding almost half of the US. Four distinct eligibility pathways were characterized, with 3 specific to CMC, and the pathways that include the presence of waiting lists were distinguished. In addition, 3 coverage types at the state level were identified, consisting of fee-for-service, Medicaid managed care, or both. Two main connections between pathways and coverage mechanisms for CMC were described.

**Conclusions and Relevance:**

The findings of this qualitative study suggest that state patterns in Medicaid eligibility and coverage for CMC have implications for access, including some states with substantial waiting periods for these families. Future work is needed to understand the implications of these differential Medicaid medical need–based eligibility pathways and subsequent coverage mechanisms on use of health care resources and expenditures, as well as considerations regarding challenges families of CMC face due to state-by-state variation.

## Key Points

**Question:**

Are there patterns in state-by-state variation in Medicaid eligibility and coverage for children with medical complexity?

**Findings:**

In this qualitative study of 43 informants, 4 distinct Medicaid eligibility pathways for children with medical complexity were identified: 3 based on medical need and 1 based on income, and 3 of which do not have waiting lists. Three state-level Medicaid coverage mechanisms for children with medical complexity were also identified.

**Meaning:**

These findings suggest that Medicaid eligibility and coverage for children with medical complexity vary substantively by state and that such variation has the potential to cause health equity concerns, especially if children move across state lines.

## Teaser

This qualitative study characterizes eligibility and Medicaid coverage focusing on medical need–based eligibility for children with medical complexity, with a focus on state-level variation.

## Introduction

Children with medical complexity (CMC) have at least 1 complex chronic condition resulting in functional limitations, family-identified health care needs, and dependence on medical technology.^1^ Families of CMC report high out-of-pocket expenses to pay for their children’s therapies and interventions,^2^ while still experiencing persistent unmet health care needs^3^ and concerns about the adequacy of employer-based insurance.^4^ Given these challenges, paths have been created for CMC to qualify for Medicaid in addition to their parents’ employer-based insurance, based on medically related criteria.^5^

Compared with the clarity of income-based Medicaid eligibility criteria, many criteria related to medical need–based eligibility vary across states and are largely descriptive in nature. Although information is available online,^6,7^ time spent navigating can lead to delays in Medicaid enrollment and benefits, causing undue stress for families.^6,8,9,10,11^ Medical need–based eligibility pathways include the medically needy provision, in which CMC qualify for Medicaid if their medical expenses reduce their household income below the categorical eligibility level in their state (eTable in the Supplement).^6^ Second, Katie Beckett (KB) waivers, also known by the Tax Equity and Fiscal Responsibility Act of 1982 (TEFRA) waivers, were established to cover the cost of institutional level care in the home setting.^12^ Third, Section 1915 waivers have the 1915(c) waiver focused on home- and community-based services (HCBS).^13^

The HCBS waivers target a specific population or diagnoses, with criteria varying by state and largely used by adults. Nationwide, 25 medically fragile waivers and 12 technology-dependent waivers are available.^14^ Previous research^10^ has highlighted differences in the number of available HCBS waivers per state, whether they cover children alone, children with medical technological needs or complex behavioral health needs, and what services are covered. After enrolling, CMC are then provided with a Medicaid coverage mechanism. Details about how coverage is operationalized—such as covering subspecialty care or specialized equipment—under differing eligibility definitions remain largely unknown.

After enrollment in Medicaid, CMC receive coverage through state-based programs, including enrollment in a Medicaid managed care (MMC) or a fee-for-service (FFS) plan. Which plan CMC are placed in affects what services are provided and covered, including therapies or home health nursing. An additional complication is whether and how CMC are enrolled in MMC organizations for the administration of benefits.^9,15,16^ Understanding coverage available to CMC is important, because coverage affects what services are covered such as preventive care, therapy, and equipment. This knowledge can inform policy advocacy regarding coverage and insurance adequacy for CMC.

Medicaid eligibility influences access to services for CMC, yet state-level eligibility and subsequent coverage differences have not been well characterized.^17^ Although most children enroll in Medicaid through categorical eligibility, in this study we set out to characterize eligibility and coverage focusing on medical need–based eligibility for CMC using qualitative methods, with a focus on state-level variation.

## Methods

### Study Design and Ethical Considerations

We conducted a qualitative study of semistructured key informant interviews with state Medicaid officials following the Standards for Reporting Qualitative Research (SRQR) reporting guideline for qualitative research.^18^ The institutional review board at Ann & Robert H. Lurie Children’s Hospital of Chicago, Illinois, determined this project to be exempt from review because it did not constitute human participant research.

### Key Informant Interviews

The interview guide was developed based on review of the literature and information available on state Medicaid websites regarding health care eligibility and coverage of CMC. The guide subsequently was reviewed by individuals with expertise in health care policy and health services research, then iteratively modified during interviews to accommodate new information such as highlighting nuances in coverage that included state identification of special populations based on needs in their state.^19^

State Medicaid officials were recruited from January 1, 2020, through January 31, 2021, using previously described snowball sampling to assess state-level policy differences.^20^ First, all listed Medicaid directors in the 50 states and Washington, DC, were emailed a recruitment invitation asking if they or a designee with expertise in and/or responsibility for child-focused programs would participate in an interview. Contact was attempted as many as 4 times. If we did not receive a response from a state, we contacted state-based child policy experts and asked for state Medicaid contact information or connections. We then reached out to these connections with a recruitment email and followed the same 4-attempt recruitment protocol. Interviews were conducted remotely via telephone or secure web platform; 23 interviews were conducted by the primary investigator (J.D.K.), and 1 was conducted by a trained research assistant.

### Data Analysis

Interviews were professionally transcribed verbatim. After transcription, documents were uploaded and analyzed using qualitative software (Dedoose, version 8.3.45 [SocioCultural Research Consultants, LLC]). Two team members (including J.D.K.) first independently created a code list focusing on non–income-based eligibility pathways and each mechanism’s coverage. Once initial coding was complete, the 2 lists were compared and reconciled. A compiled code list was then discussed within the larger research group until the codebook was finalized.^19,21,22^ Codes were then organized into clusters of content until no new organization of codes were identified.

After content analysis, we composed a summary of each interview and shared it with each participating official, who was given the option to review and make corrections to the content. We synthesized the findings to describe and then characterize 2 key outcomes: the typical eligibility determination and enrollment processes experienced by CMC by state and related mechanisms of coverage. Eligibility pathways were defined as available options by which CMC can enroll in Medicaid and receive Medicaid benefits. Coverage mechanisms were defined as the coverage process used to pay for health care for CMC, either FFS or MMC. Eligibility and coverage were then compared across states to inform general categorizations.

## Results

### State Demographics

Interviews were conducted from February 1, 2020, to March 1, 2021, with 1 to 4 individuals per state. A total of 43 informants from 23 states and Washington, DC (hereafter referred to as states), participated. Interviewees included Medicaid directors, policy analysts, directors of home health programs, program administrators, and physicians with state-level roles. Our sample included geographic state diversity representing all major census regions of the US (West [n = 8], Midwest [n = 5], Northeast [n = 4], and South [n = 7]).^23^

### Eligibility Pathways

**Figure 1.**
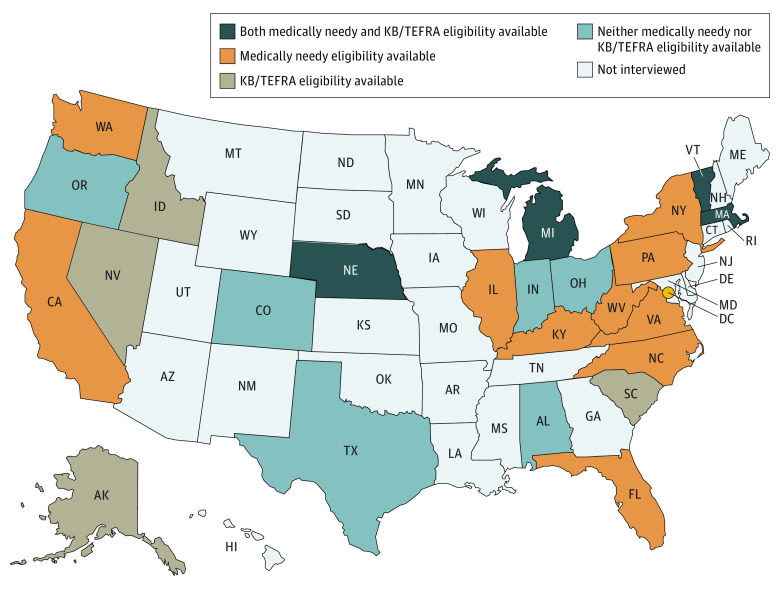
Map of Participating States That Have a Medically Needy or a Katie Beckett/Tax Equity and Fiscal Responsibility Act (KB/TEFRA) Eligibility Option

We identified 4 pathways of state eligibility available to CMC: income-based eligibility, and 3 additional pathways uniquely available based on medical diagnoses or need for CMC. These included (1) medically needy spend-down eligibility, (2) KB/TEFRA eligibility, and (3) HCBS waivers (Table and eTable in the Supplement). Of these 3 CMC-specific eligibility pathways, HCBS waiver pathways have limited enrollment and corresponding waiting lists. We further sorted states based on whether they offer medically needy spend-down eligibility, the KB/TEFRA eligibility pathway, both options, or neither option besides income eligibility available for CMC without waiting lists (Figure 1). Among the states that participated in our study, states most commonly had medically needy eligibility and did not have KB/TEFRA eligibility pathways. The least common were states that had both medically needy and KB/TEFRA eligibility pathways available.

**Table.** Summary of State Eligibility and Coverage Themes by States

State	Pathway to enroll CMC into Medicaid^a^			Special populations^b^	Dual private insurance enrollment	Waiting lists	Medicaid mechanism type	Managed care carve-outs^c^
	Medically needy	KB/TEFRA waiver	HCBS waivers					
Alabama	No	No	Yes	No	Yes	Yes	FFS	No
Alaska	No	Yes	Yes	No	Yes	Yes	FFS	No
California	Yes	No	Yes	Yes	Yes	Yes	MMC	Yes
Colorado	No	No	Yes	Yes	Yes	No	FFS	No
Florida	Yes	No	Yes	Yes	Not answered	Yes	MMC and FFS	Yes
Idaho	No	Yes	Yes	Yes	Yes	No	FFS	No
Illinois	Yes	No	Yes	Yes	Yes	No	MMC and FFS	Not answered
Indiana	No	No	Yes	Yes	Yes	Yes	MMC and FFS	Yes
Kentucky	Yes	No	Yes	Yes	Yes	Yes	MMC and FFS	Yes
Massachusetts	Yes	Yes	Yes	Yes	Yes	No	MMC and FFS	Yes
Michigan	Yes	Yes	Yes	No	Yes	Yes	MMC and FFS	Yes
Nebraska	Yes	Yes	Yes	No	Yes	Yes	MMC and FFS	Yes
Nevada	No	Yes	Yes	No	Yes	Yes	MMC and FFS	Yes
New York	Yes	No	Yes	No	Yes	No	MMC	Yes
North Carolina	Yes	No	Yes	No	Yes	Yes	MMC and FFS	No
Ohio	No	No	Yes	No	Not answered	Yes	MMC and FFS	No
Oregon	No	No	Yes	No	Yes	Yes	MMC	No
Pennsylvania	Yes	No	Yes	No	Yes	Yes	MMC and FFS	No
South Carolina	No	Yes	Yes	No	Yes	No	MMC and FFS	No
Texas	No	No	Yes	No	Yes	Yes	MMC	Yes
Vermont	Yes	Yes	Yes	No	Yes	No	MMC and FFS	Not answered
Washington, DC	No	Yes	Yes	No	Yes	No	MMC and FFS	No
Washington	Yes	No	Yes	Yes	Yes	Yes	MMC	Yes
West Virginia	Yes	No	Yes	Yes	Yes	No	MMC and FFS	Yes

Abbreviations: CMC, children with medical complexity; FFS, fee-for-service; HCBS, home- and community-based services; KB/TEFRA, Katie Beckett/Tax Equity and Fiscal Responsibility Act; MMC, Medicaid managed care.

^a^ Includes income-based eligibility (traditional Medicaid).

^b^ Includes varying groups across states (eg, social security income, neonatal abstinence syndrome, Native American tribes, low birth weight, and foster care).

^c^ Services such as long-term services and supports or dental care are carved out of MMC and covered by FFS.

### Enrollment Processes

Enrollment duration varied; most states used a 1-year enrollment period, with annual recertification processes. For children enrolled through medically needy eligibility, families must spend down to a specific threshold determined by states, which is verified through submission of medical expenses every 1 to 6 months. State officials in South Carolina and Pennsylvania, for example, pointed out that enrollment for the medically needy and KB/TEFRA pathways may have determination periods (ie, time spent waiting while medical diagnoses or family asset information is verified and applications are processed). Another source of delay that South Carolina described is the inability to enroll participants admitted to the hospital until they are discharged and back in their home or a community-based setting. Both delays, however, are not considered true waiting periods because they are not based on limitations to the number of children enrolled.

States provided various rationales for not participating in a medically needy eligibility program, including the financial burden that it can place on families having to meet their spend-down amounts. Colorado instead has a “buy-in” option for families that make less than 300% of the federal poverty level (Box). Indiana similarly explained not having this pathway due to the paperwork burden on families (Box). Of the states with medically needy eligibility, many reported that it was not commonly used for children, and that most children enrolled through an available HCBS waiver instead.

Box. **Illustrative Quotations of State Medicaid Experts**

**Theme: Eligibility Pathways**

**Subtheme: Not Having a Medically Needy Eligibility Provision**

**Colorado:** This decision was made because “we didn’t want to make families have to do that [medical] spending because a lot of them have to sell their house or their car to do that spending. So, we wanted to be sure that they had a way in [to Medicaid access] without having to do that spend down.”
**Indiana**: The medically needy eligibility provision “can be extremely cumbersome to administrate and burdensome for [applicants’ families].”

**Subtheme: Special Populations and Private Insurance**

**Massachusetts:** “We do an insurance investigation on every single new member, and if they have access to qualifying employer-sponsored insurance, we will require them to enroll and we’ll pay their premium.”
**Nevada:** “We actually encourage and require them to keep their [private insurance] because Medicaid should always be the care of last resort.”

**Subtheme: Simplification of Waiver Process**

**New York:** “Previous to April of 2019 we had 6 children’s waivers and in April of 2019 combined all those waivers into 1 children’s waiver and there was a number of reasons for that but the biggest reason was we didn’t want to have wait lists.”

**Theme: Coverage Mechanisms**

**Subtheme: CMC Are Placed Into a Specific Coverage Plan**

**South Carolina**: Children with medical complexity are placed into an MMC plan to help CMC “get more intense [nurse-provided] care coordination that would definitely benefit the child and their family.”
**Washington, DC:** “Ninety percent of kids are enrolled in managed care, so that’s the vast majority of them, but there are some disabled children who are in our fee-for-service program and then we also have a special needs plan as part of our managed care program.”

Abbreviations: CMC, children with medical complexity; MMC, Medicaid managed care.

### Special Populations and Private Insurance

Ten states reported having waivers or automatic eligibility for special pediatric populations or those with special diagnoses to enroll in Medicaid that were separate from income eligibility (Table). These special populations ranged from immigrant children to infants with neonatal abstinence syndrome to infants in foster care.

State Medicaid programs also allow children to have private or employer-based insurance in addition to their Medicaid coverage. In these instances, Medicaid is the payer of last resort, meaning it covers what private insurance does not. In these cases, officials in both Massachusetts and Nevada explained that they encourage families to keep their private insurance (Box).

### Waivers and Waiting Lists

The HCBS waivers are unique in that states are allowed to impose enrollment caps as a method of managing growth and expenditures, which therefore have the potential to lead to waiting lists for eligible individuals. Resulting waiting lists for HCBS waivers are common: all states interviewed had HCBS waivers available to CMC, with 15 (63%) reporting children currently on a waiting list for enrollment (Table). New York simplified to 1 waiver, resulting in no children on a waiting list (Box). Officials in other states reported that the time that CMC spend on HCBS waiver waiting lists may extend to several years.

### Coverage Mechanisms

**Figure 2.**
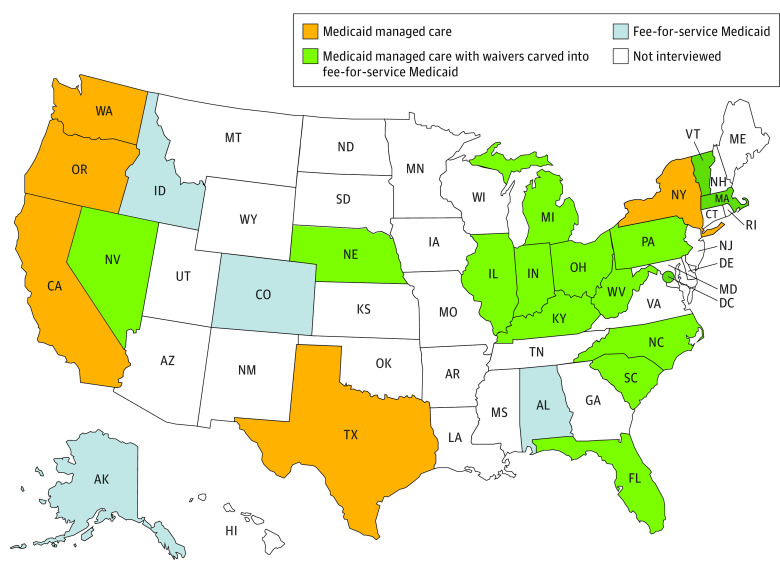
Map of Coverage Mechanisms for Children With Medical Complexity by State

We identified 3 state approaches to coverage for CMC: MMC, FFS, and a combination of both. Of the states interviewed, 4 used FFS Medicaid exclusively; 5 used MMC only, including for CMC; and 15 used a combination of MMC in general for Medicaid coverage, but FFS for specific populations of CMC (Figure 2).

In some states, CMC who are eligible through HCBS waivers or the KB/TEFRA pathway are placed into the same pool as all other children enrolled in Medicaid, and therefore receive the same coverage. In other states, CMC enrolled through HCBS waivers are placed in a specific plan. In South Carolina, children enrolled through the Medically Complex Children’s Waiver are placed in an MMC plan to help receive specific benefits (Box). In Kentucky, CMC enrolled through waiver services are placed into FFS plans while other children are in MMC plans.

The overlap of states’ eligibility provisions and coverage structures varied. Among the 4 states that exclusively used FFS Medicaid, none had medically needy eligibility, and 2 of these 4 states offered KB/TEFRA eligibility as an option. The 4 states that offered both medically needy and KB/TEFRA eligibility all have a combination coverage model, with CMC eligible through HCBS waivers or some waiver-based services being carved into FFS Medicaid. None of the states that used KB/TEFRA eligibility used exclusively MMC.

### Summary of the Medicaid Eligibility and Coverage Mechanisms for CMC

**Figure 3.**
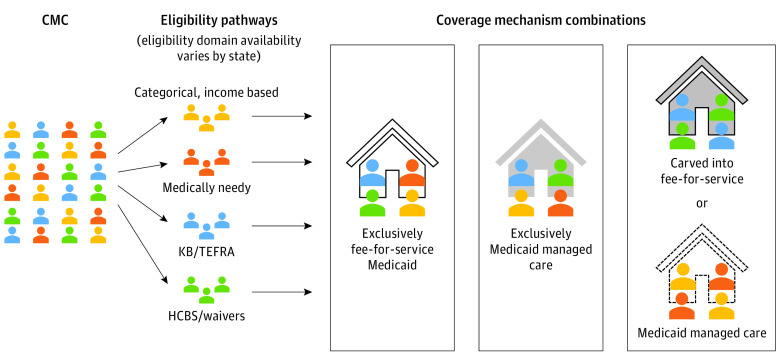
Representation of Medicaid Eligibility Pathways Available to Children With Medical Complexity (CMC) and Subsequent Coverage Mechanisms HCBS indicates home- and community-based services; KB/TEFRA, Katie Beckett/Tax Equity and Fiscal Responsibility Act.

Children with medical complexity have multiple eligibility pathways for Medicaid, depending on existing policies and programs (Figure 3). Of the 4 identified eligibility pathways, 3 did not having waiting lists associated with them: categorical, medically needy, and KB/TEFRA eligibility.

After eligibility determination, coverage varied across states in ways that are not strictly determined by eligibility: each state Medicaid program placed CMC beneficiaries into a plan with a mechanism of coverage that determines their benefits (Figure 3). In one state, CMC who have Medicaid eligibility through a medically needy path may be placed in an MMC plan, whereas in another state special provisions may require eligible CMC to receive FFS coverage. Ultimately, the eligibility pathways available to CMC in each state, and the subsequent coverage mechanism based on each state’s coverage rules, may affect ultimate patterns of use of health care resources by CMC.

Two general patterns emerged when characterizing state eligibility pathways and coverage mechanisms in tandem. In the 4 states that offer all eligibility pathways (Massachusetts, Michigan, Nebraska, and Vermont), all 4 cover children using a mix of MMC and FFS. In the 6 states that have only HCBS waivers available in addition to categorical eligibility, 4 use only MMC or only FFS (Figures 1 and 2).

## Discussion

In this multistate study, we characterized differences in state-level patterns of medical need–based eligibility for Medicaid and coverage mechanisms for CMC. We attempted to illuminate key differences across states reflecting policies that may help families and clinicians navigate the Medicaid system for CMC. However, state-by-state differences may confuse families and lead to delays in coverage and access to care.

Eligibility options available to CMC may have profound effects on securing Medicaid coverage, specifically related to HCBS waivers, their enrollment caps, and commonly associated waiting lists.^24^ Although HCBS waivers vary by state, they must remain cost-neutral, leading states to impose enrollment caps to manage expenditures.^24^ This means that in a state without the medically needy or KB/TEFRA eligibility provisions (which was the second most common eligibility pathway combination among interviewed states), CMC may not be eligible for Medicaid services without obtaining an HCBS waiver and potentially entering a subsequent waiting period. Some states have faced rising rates of children needing HCBS, contributing to growing waiting lists.^14^ States with medically needy provisions might view such eligibility as critically important for CMC. In states without such provisions, eligibility for CMC will depend on alternative eligibility avenues such as a buy-in option or may otherwise be constrained in offering coverage because of state priorities regarding control of Medicaid expenditures.

For example, variation across states could lead to a child with medical complexity who is in a non–income-eligible family but has medically needy eligibility gaining access to Medicaid coverage within months, whereas a child with an identical family income and health status living in another state with the HCBS waiver pathway may wait years for coverage. If an enrolled child’s family were to move, the child may have to join a waiting list in their new state of residence and potentially wait years before reaccessing Medicaid and returning to the same level of coverage as in their previous state of residence.^25^ Our finding of geographic inequity related to health policy is consistent with other research.^10,26,27^ In our study, we found that state-by-state variability poses health equity concerns as families face decisions about whether to move across state borders and thereby gain or lose benefits available to CMC.^28^

Due to differences across states regarding CMC enrollment in Medicaid, and the resulting complexity and confusion, grassroots online resources have been created by and for families to help navigate what eligibility pathways are available for CMC.^7^ In addition, families and clinicians face multifaceted challenges navigating the complex health care system for CMC, including having sufficient private duty nurses, shortages and other difficulties with equipment supplies, and problems in finding therapists.^1,29,30^ Although government-sponsored systems such as Title V are in place to reduce challenges, they cannot coordinate services that are not covered.

Nationwide, a trend has shifted toward MMC coverage for children.^31^ In 2020, it was reported that there had been an increase in enrollment in MMC for children with special health care needs compared with 3 years prior.^32^ Medicaid managed care includes care coordination and the patient-centered medical home as basic tenets, which are important services for CMC. Moreover, policy experts have suggested that MMC enrollment of CMC can cause delays in care during transition from FFS owing to changes in clinician networks and formularies.^2,3^ In addition, 1 study reported MMC-driven denial of services.^33^ Therefore, although MMC is conceptually consistent with the goals of care for CMC, pragmatic realities may obstruct or delay the care that CMC receive, and these patients’ experiences merit further research in the MMC environment.

When connecting state Medicaid eligibility with coverage for CMC, 2 general patterns emerged. One group of states have multiple routes of eligibility and are also more likely to combine MMC with FFS approaches for different populations. This more abundant set of options is potentially more complex for CMC to navigate. Another group of states offers a more restricted set of eligibility options for CMC and tends to provide a single-approach-fits-all (eg, FFS) for coverage. Fewer eligibility options may translate into functional restrictions (eg, waiting lists) for CMC; although the system is simpler to navigate, enrollment may be available for fewer CMC at a given time, and coverage structures may not change with CMC needs. Which of these patterns a state chooses may reflect their attempts to manage enrollment growth and expenditures.

### Limitations

This study should be interpreted within the context of its limitations. The qualitative data collected are representative of the 24 states in which we obtained interviews; the generalizability of the study findings to other states is unknown. We describe the current state of eligibility and coverage for CMC and acknowledge that, if current trends persist, most children may be covered through a managed care entity. Because we were unable to interview sources in all 50 states, we did not learn all possible mechanisms for Medicaid eligibility and coverage. This limitation is due in part to the COVID-19 pandemic, which may have limited the availability of Medicaid personnel. Despite this constraint, states in which representatives were interviewed did represent a geographically broad swath of the country. Additionally, the study’s information is limited to the knowledge base of the interviewee or the information they were able to provide us, and inaccuracies are possible. However, our findings reflect the most comprehensive, publicly available information we could identify on this subject, available from persons who agreed to be interviewed on this topic for their state programs.

## Conclusions

The findings of this qualitative study suggest that state-by-state variations in Medicaid eligibility and coverage for CMC have implications for access, including some states with substantial waiting periods. State-by-state variability deserves further investigation regarding how CMC ultimately use care differently across states and how their health outcomes vary as a result. For states that do not have a medically needy or a KB/TEFRA option, investigation is warranted to determine whether these states rely more heavily on HCBS waivers and therefore have longer wait times and less use of health care resources for CMC. These findings offer new insights and raise additional questions that could inform future advocacy efforts regarding policy changes to address the health needs of CMC through Medicaid coverage, acknowledging state-by-state differences that may persist over time.

## Notes

Supplement. **eTable.** Key Terms and Definitions
