# Supplementary material for: Characteristics of Medicaid Policies for Children With Medical Complexity by State: A Qualitative Study
Source: JAMA Netw Open. 2022 Oct 31;5(10):e2239270. doi: 10.1001/jamanetworkopen.2022.39270 (PMC9623434; doi:10.1001/jamanetworkopen.2022.39270)
Supplement: Supplement. — eTable. Key Terms and Definitions [file jamanetwopen-e2239270-s001.pdf]

Supplementary Online Content

Kusma JD, Davis MM, Foster C. Characteristics of Medicaid policies for children with medical complexity by state. *JAMA Netw Open*. 2022;5(10):e2239270. doi:10.1001/jamanetworkopen.2022.39270

eTable. Key Terms and Definitions

This supplementary material has been provided by the authors to give readers additional information about their work.

© 2022 Kusma JD et al. *JAMA Network Open*.

eTable. Key Terms and Definitions

Key Term	Definition
Categorical Medicaid Eligibility	Eligible for Medicaid coverage based on household income below state-based income threshold, typically determined by the family's Modified Adjusted Gross Income (MAGI).
Fee-for-service	Fee-for-service is also known as traditional Medicaid. This is where a provider is paid for each service that is provided to a beneficiary.
Medicaid Managed Care	Managed care is where an organization is paid a set amount per beneficiary enrolled in their organization.
Waiver	Waivers are a mechanism to target specific populations or diagnoses, with criteria that vary by state. Waivers can impose enrollment caps to control expenditures, and therefore many have waiting lists.<br><br>One example of a waiver is the 1915c waivers focus on Home and Community Based Services and are an area of focus for this project. There are other waivers, including 1115 waivers, which are innovations to the state Medicaid program. Waivers can be diagnosis based, for example children can enroll due to medical technology needs or due to complex behavior health needs.
Home and Community Based Services (HCBS)	HCBS provides opportunities for home or community services rather than institutional based. A range of services can be included from home health care and durable medical equipment. Services are often provided via 1915c waivers.
Katie Beckett Waiver	This waiver is also known by the name of the act that enabled the waiver, the Tax Equity and Fiscal Responsibility Act of 1982 (TEFRA). It is different from other waivers in that it has no enrollment caps.

© 2022 Kusma JD et al. *JAMA Network Open*.
